# Three Independent Mesial Canals in a Mandibular Molar: Four-Year Followup of a Case Using Cone Beam Computed Tomography

**DOI:** 10.1155/2013/891849

**Published:** 2013-04-15

**Authors:** Adrianne Freire de Paula, Manoel Brito-Júnior, Alex Carvalho Quintino, Carla Cristina Camilo, Antônio Miranda Cruz-Filho, Manoel Damião Sousa-Neto

**Affiliations:** ^1^Department of Dentistry, State University of Montes Claros, Avenue Rui Braga s/n, 39401-089, Montes Claros, MG, Brazil; ^2^Department of Restorative Dentistry, Faculty of Dentistry, University of São Paulo, Avenue Bandeirantes 3900, 14040-900, Ribeirão Preto, SP, Brazil

## Abstract

Endodontic treatment of mandibular molars is challenging because of variable root canal morphology. The nonsurgical endodontic management of a mandibular first molar presenting an independent middle mesial canal is reported. After coronal access, additional clinical inspection of the mesial canals' orifices and their interconnecting groove using an endodontic explorer and 4.5× loupes enabled the identification of the middle mesial canal orifice. All root canals were chemomechanically prepared and filled. The tooth was asymptomatic and functional after 4 years of followup. Cone beam computed tomography (CBCT) images revealed normal periapical status and three-dimensional (3D) anatomical aspects of the root canal system.

## 1. Introduction

Endodontic treatment of mandibular molars is a constant challenge to the clinician because the number of root canals is highly variable. The root canal system of these teeth consists of fins, accessory, secondary, and lateral canals, as well as transverse anastomosis [1]. Since chemomechanical preparation is a critical step for a successful endodontic outcome, a thorough root canal debridement including additional canals must be achieved [2]. Thus, a better understanding of tooth morphology favoring the search for root canals is essential to attain this purpose.

The first mandibular molars typically have two roots, one mesial with two root canals (mesiobuccal and mesiolingual) and another distal, which contains one or two root canals [1, 2]. Root canal configuration with two distal canals varies from 20% to 46% in different populations [3, 4]. The identification of three [5, 6] or four [7] canals in the mesial root or three canals in the distal root [8] is still anatomical variations reported in the literature.

Regarding the presence of the middle mesial (MM) canal having an independent trajectory in the mesial root of first mandibular molars, a literature search revealed few cases reported over the last decades (Table 1). In addition, the most cases [9–15] were identified by periapical radiographs, which have limitations in describing 3-dimensional (3D) roots and root canal anatomy. Thus, this case report describes the endodontic management of three independent mesial canals in a first mandibular molar where cone beam computed tomography (CBCT) was used at the 4-year followup. 

## 2. Case Report

**Figure 1:**
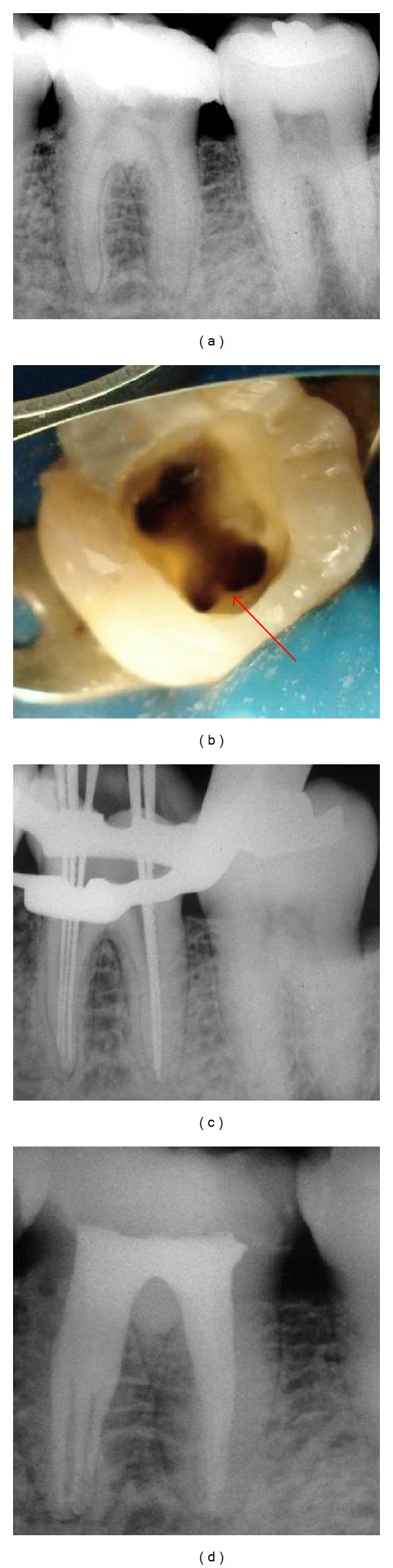
(a) Preoperative radiograph. (b) Clinical view of root canal entrances including a middle mesial canal (arrow). (c) Radiograph for working length determination showing three independent mesial canals. (d) Postoperative radiograph.

 A 42-year-old male patient with noncontributory medical history was referred for endodontic treatment of the left first mandibular molar. The clinical examination of the tooth revealed no swelling or sinus tract and a slight response to percussion and palpation. The tooth did not respond to thermal or electric pulp tests. A periapical radiograph revealed a deep distal carie and a slight widening of the apical periodontal ligament in the distal and mesial roots (Figure 1(a)). Based on the clinical and radiographic findings, the diagnosis was established as a pulp necrosis. A treatment plan, including nonsurgical root canal therapy of tooth, was presented to the patient. The patient consented to the proposed treatment. 

During the first session, local anesthesia was administered, and a rubber dam was applied. Upon access opening, four well-defined root canal orifices were located using an endodontic explorer (Odous, Belo Horizonte, MG, Brazil) on the pulpal floor, two orifices for the distal root, and two separate orifices for the mesial root. The subpulpal groove of the mesiobuccal root was located after removing the excess dentin using an ultrasonic device (Gnatus, Ribeirão Preto, SP, Brazil) with an ultrasonic tip (TU17; Trinity, São Paulo, SP, Brazil). The tip of the explorer attached in the middle mesial canal orifice, which was located closer to the mesiobuccal canal (Figure 1(b)). A 4.5× binocular loupe (Bio-Art Equipamentos Odontológicos, São Carlos, SP, Brazil) was used to facilitate this procedure. The radiograph taken to determine the working lengths showed three independent mesial canals (Figure 1(c)). All root canals were prepared in a crown-down method using ProFile Taper 04/06 rotary NiTi instruments (Maillefer-Dentsply, Ballaigues, Switzerland). Preparation was always performed under irrigation with 2.5% sodium hypochlorite solution followed by smear layer removal with a 14.3% EDTA solution (pH 7.2) for 3 minutes. Afterward, the root canal was dried with paper points and filled with calcium hydroxide paste (Calen, SS White, Rio de Janeiro, RJ, Brazil) using a lentulo spiral. The cavity entrance was closed with a noneugenol temporary filling material (Coltosol, Vigodent, Rio de Janeiro, RJ, Brazil).

 The patient returned for a second appointment 10 days later. The temporary restoration was removed under local anesthesia and rubber dam. The root canals were irrigated with 2.5% sodium hypochlorite, and the intracanal dressing was removed, stirring with a size 15 K-file (Maillefer-Dentsply, Ballaigues, Switzerland), followed by a final rinse with a 14.3% EDTA. The canals were dried and filled with a zinc oxide-eugenol-based sealer (Endofill, Dentsply, Petrópolis, RJ, Brazil) and nonstandardized gutta-percha cones (Odous, Belo Horizonte, MG, Brazil) using cold lateral condensation. A temporary filling was placed and a postoperative radiograph was taken to assess the quality of obturation (Figure 1(d)). The patient was referred for appropriate coronal restoration.

**Figure 2:**
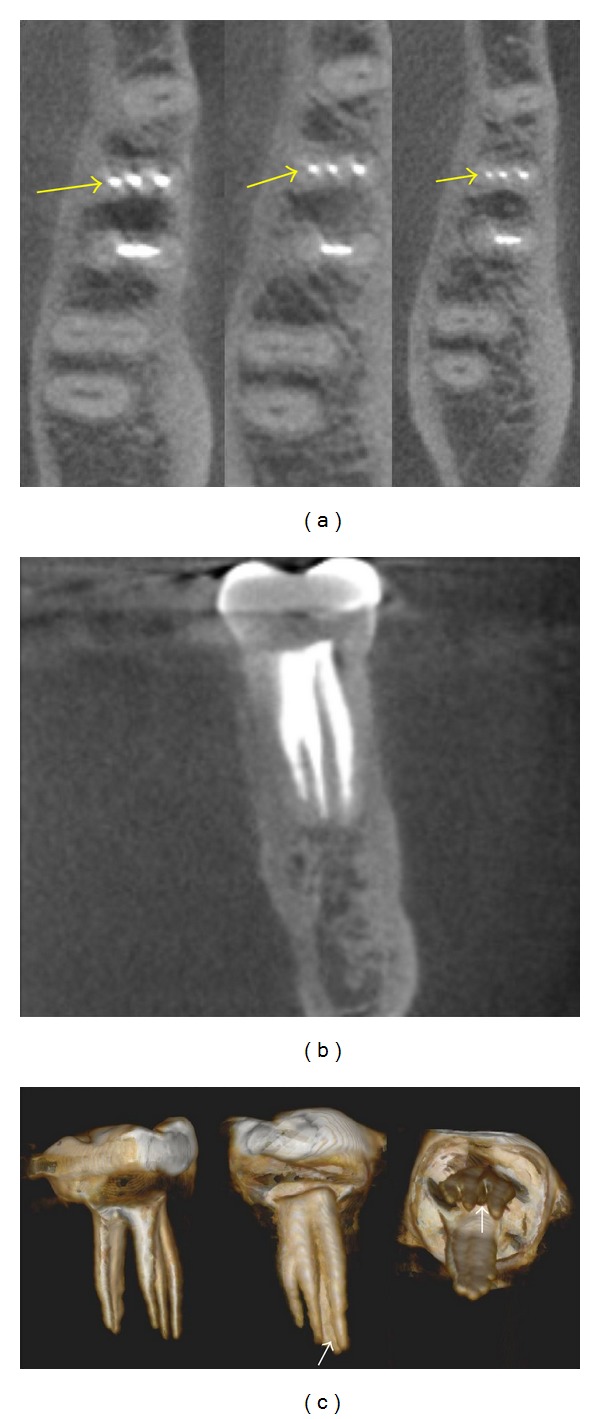
(a) Axial CBCT slices on coronal, middle and apical root sections (arrows) showing three independent mesial canals. (b) Coronal CBCT view of mesial root with three filled root canals. (c). 3-Dimensional rebuild illustrating the internal configuration including a filled isthmus between the mesiobuccal and middle mesial canals (arrows).

The tooth was asymptomatic and functional after 4 years of followup. The patient was informed about the possibility of CBCT examination, which was unavailable at the time of the initiation of treatment. It was explained that CBCT could be useful to check three-dimensionally the endodontic treatment performed. Thus, CBCT images focused on tooth number 19 were obtained after informed consent of the patient. Axial (Figure 2(a)) and sagittal (Figure 2(b)) slices of 0.076 mm thickness using the CBCT scan (Kodak 9000 3D, Carestream Health, Inc., Rochester, NY, USA) revealed normal periapical status and three independent filled root canals in the mesial root. A three-dimensional rebuild performed from the CBCT images showed in detail the internal configuration of the all filled root canals (Figure 2(c)). 

## 3. Discussion 

The present case report describes the treatment of a first mandibular molar with three mesial root canals. A systematic review conducted by de Pablo et al. [16] reported that the overall incidence of this anatomical morphology was 2.6%. They also showed that the confluence of the MM canal with the mesiobuccal or mesiolingual canals having a common apical termination was more frequently observed than a configuration including three distinct, separate apical foramina [16]. This last condition, as presented in this report, is recognized as 3-3 configuration (type 8) by using Vertucci's classification [1]. The entrance of the MM canal was located closer to the mesiobuccal canal, which corresponds to an incidence of 25% according to a previous investigation [17]. 

The detection of root canal orifices may be influenced by the complexity of the internal anatomy and operator experience [18]. Furthermore, a magnified view of the operating field using dental microscopic or surgical loupes increases the chance of locating hidden canals [5, 7, 17, 18]. The use of a microscope or the use of loupes by practicing endodontists was equivalent in searching for additional canals in first mandibular molars [17]. The endodontic treatment of the present case was performed by an experienced endodontist familiar with the use of loupes for more than 10 years. In addition, a careful examination of the pulpal floor with an endodontic explorer and the use of an ultrasonic tip to remove the dentin coverage over the middle mesial canal orifice were important procedures to ensure the successful treatment [5]. 

The existence of an MM canal is sometimes observed in angled horizontal radiographs. However, molar cases may require 3D diagnostic images to improve the assessment of root canal systems [6, 8]. In this situation, CBCT provides additional information to detect extra canals. In a vitro study, de Toubes et al. [5] demonstrated that CBCT was an accurate method of identifying accessory mesial canals in mandibular first molars. CBCT also was a helpful imaging tool used by La et al. [6] to detect clinically a challenging case of an independent middle mesial canal. 

The presence of three mesial canals that were independent throughout their course in the root was visualized on 3D CBCT images during the follow-up period in the present case. The Kodak 9000 3D used in this report is categorized as a small-volume CBCT based on its scan field of view (FoV), which covers only a few teeth or one jaw. It has been reported that small FoV provides better image quality with lower effective radiation when compared with medium and large FoV devices [19]. In addition, cone beam reconstructions at 0.076 mm obtained with Kodak 9000 3D had a minimal discrepancy compared to histologic sections of extracted teeth with multiple root canal anatomy [20]. The reconstructed images of the root-filled tooth in this particular case showed a similar length of MM and mesiobuccal canals, as well as a filled isthmus between these two canals. The mesiolingual canal presented the smaller dimensions. 

In summary, this report corroborates the presence of three independent mesial root canals in a mandibular first molar. Moreover, the details of this internal configuration with filled root canals were displayed on 3D CBCT images. 

## Figures and Tables

**Table 1:** Case reports on three independent mesial canals of first mandibular molar.

Author, year	Evaluation technique	Number of teeth
La et al., 2010 [6]	CBCT	1
Yesilsoy et al., 2009 [9]	PA radiographs	1
Mortman and Ahn, 2003 [10]	PA radiographs	1
Holtzman, 1997 [11]	PA radiographs	1
Ricucci, 1997 [12]	PA radiographs	1
Jacobsen et al., 1994 [13]	PA radiographs	1
Bond et al., 1988 [14]	PA radiographs	1
Martinez-Berna and Badanelli, 1985 [15]	PA radiographs	1

CBCT: cone beam computed tomography, PA: periapical.
